# Homocysteine Induces Apoptosis of Human Umbilical Vein Endothelial Cells via Mitochondrial Dysfunction and Endoplasmic Reticulum Stress

**DOI:** 10.1155/2017/5736506

**Published:** 2017-05-28

**Authors:** Zhimin Zhang, Congying Wei, Yanfen Zhou, Tao Yan, Zhengqiang Wang, Wei Li, Lianyou Zhao

**Affiliations:** ^1^Department of Cardiology, Tangdu Hospital of the Fourth Military Medical University, Xi'an, China; ^2^Department of Cardiology, Xian Yang Central Hospital, Xi'an, China; ^3^Department of Cardiology, Shaanxi Provincial Chinese Traditional Medicine Hospital, Xi'an, China; ^4^Department of Pharmacy, Tangdu Hospital of the Fourth Military Medical University, Xi'an, China

## Abstract

Homocysteine- (Hcy-) induced endothelial cell apoptosis has been suggested as a cause of Hcy-dependent vascular injury, while the proposed molecular pathways underlying this process are unclear. In this study, we investigated the adverse effects of Hcy on human umbilical vein endothelial cells (HUVEC) and the underlying mechanisms. Our results demonstrated that moderate-dose Hcy treatment induced HUVEC apoptosis in a time-dependent manner. Furthermore, prolonged Hcy treatment increased the expression of NOX4 and the production of intracellular ROS but decreased the ratio of Bcl-2/Bax and mitochondrial membrane potential (MMP), resulting in the leakage of cytochrome c and activation of caspase-3. Prolonged Hcy treatment also upregulated glucose-regulated protein 78 (GRP78), activated protein kinase RNA-like ER kinase (PERK), and induced the expression of C/EBP homologous protein (CHOP) and the phosphorylation of NF-κb. The inhibition of NOX4 decreased the production of ROS and alleviated the Hcy-induced HUVEC apoptosis and ER stress. Blocking the PERK pathway partly alleviated Hcy-induced HUVEC apoptosis and the activation of NF-κb. Taken together, our results suggest that Hcy-induced mitochondrial dysfunction crucially modulated apoptosis and contributed to the activation of ER stress in HUVEC. The excessive activation of the PERK pathway partly contributed to Hcy-induced HUVEC apoptosis and the phosphorylation of NF-κb.

## 1. Introduction

Hyperhomocysteinemia (HHcy) is an independent risk factor for cardiovascular diseases, even moderate HHcy being independently responsible for the development of premature atherosclerosis and thrombosis [1–3]. Moderate HHcy is highly prevalent in the general population and exerts an adverse effect on vascular structure and function, which has not attracted enough attention. Oxidative radical-mediated apoptosis or cellular dysfunction, which is responsible for diminished production and bioavailability of endothelial-derived NO, can cause impaired endothelial-dependent vascular reactivity [4–6]. Under physiological conditions, most of the intracellular reactive oxygen species (ROS) are generated from mitochondrial dysfunction [7, 8]. Furthermore, mitochondrial dysfunction may modulate multiple molecular and cellular signaling mechanisms contributing to the occurrence of apoptotic events. Specifically, mitochondrial dysfunction activates caspase-dependent apoptotic signaling pathways, directly resulting in cellular death. Nevertheless, whether mitochondrial dysfunction plays a crucial role in the development of Hcy-induced HUVEC apoptosis is unclear.

Currently, ER stress has attracted wide attention, because it has been found to be involved in the pathophysiology of many cardiovascular diseases, such as pressure overload [9], arteriosclerosis [10], and Hcy-induced cardiopathy [11]. Previous studies suggested that Hcy could initiate endoplasmic reticulum (ER) stress in HUVEC based on the upregulation of ER chaperones [12–14]. Nevertheless, how Hcy induces ER stress in HUVEC and whether the activation of ER stress directly induces HUVEC apoptosis are not well understood. ER stress is initiated by the accumulation of misfolded proteins in the ER lumen. Moderate stress induces the dissociation of ER resident chaperones GRP78 from a transmembrane protein PERK, enhancing the abilities of handling misfolded proteins [15, 16]. Severe stress upregulates the expression of CHOP, triggering the proapoptotic signaling pathway [17]. Previous studies suggested that ER stress and mitochondrial dysfunction coexist in many pathologic states [18–20], and there could be some relationship between ER stress and mitochondrial dysfunction. However, studies of the precise mechanisms linking ER stress and mitochondria stress are just underway. Therefore, we are interested in whether and how the two pathomechanisms interact in HUVEC exposed to Hcy.

Chronic inflammation has also been proposed as a biologic mechanism underlying Hcy-related vascular diseases [21–23]. It has been shown that the activation of NF-*κ*b plays a pivotal role in the cellular signaling mechanism for various toxicant-induced inflammations [24, 25]. Although previous studies demonstrated that various signaling pathways were responsible for the activation of NF-*κ*b in many pathologic states, the previous work has provided only a limited viewpoint regarding the signaling pathway modulating NF-*κ*b in HUVEC exposed to Hcy. Therefore, we performed the present study to examine the effects of Hcy on mitochondrial function and ER stress and analyzed the relationship between the two pathological mechanisms and HUVEC apoptosis. Moreover, the activation of NF-*κ*b in Hcy-induced HUVEC and the underlying regulating mechanism were explored.

## 2. Materials and Methods

### 2.1. Materials

Human umbilical vein endothelial cells (HUVEC) were obtained from the Cell Line Bank of the Chinese Academy of Sciences (Shanghai, China). Dulbecco's modified Eagle's medium (DMEM) and fetal bovine serum (FBS) were purchased from Gibco Technologies (Logan, UT, USA). DL-Homocysteine, tunicamycin, diphenyleneiodonium (DPI), and salubrinal were obtained from Sigma (St Louis, MO, USA). siRNA and Lipofectamine® 2000 were purchased from Invitrogen (Carlsbad, CA, USA). Antibodies against PERK (3192s), p-PERK (3179s), eIF2a (5324s), p-eIF2a (3597 s), ATF4 (11815S), caspase-3 (9662), cytochrome c (4272), and actin (12262s) were purchased from Cell Signaling Technology (Boston, MA, USA). Antibodies against GRP78 (ab21685), CHOP (ab11419), p-eNOS (76199), Bax (ab32503), and Bcl-2 (ab59348) were purchased from Abcam (Boston, MA, USA). Electrochemiluminescence (ECL) Western blotting detection reagents were obtained from Millipore (Bedford, MA, USA). Cell Counting Kit-8 (CCK-8) and DCFH-DA staining were purchased from Beyotime (Jiangsu, China). JC-1 staining, mitochondrial ROS fluorescent probe, and Annexin V-FITC/PI were obtained from Molecular Probes (Invitrogen, Milan, Italy).

### 2.2. Cell Culture and Treatment

HUVEC were cultured in DMEM containing 10% FBS, 100 U/mL penicillin, and 100 *μ*g/mL streptomycin at 37°C in a 5% CO_2_ environment. Cells that had grown to 80% confluence were used in the experiments. The cultures were treated with 2.0 mmol/L Hcy for 0, 6, 12, 24, or 36 hours. The cells were pretreated with 10.0 *μ*mol/L DPI or 20.0 *μ*mol/L salubrinal for 60 min and were cotreated with 2.0 mmol/L Hcy for 24 hours. The cells were transfected with 50 nmol/L siRNA against rat CHOP (targeting sequences for CHOP: 5′-CUAGAAAUCUGUUGCUAUG-3′) using siRNA transfection reagent according to the manufacturer's instructions. Six hours after transfection, the cells were replanted on culture dishes, followed by the addition of Hcy for 24 hours. After treatment, the cells were immediately washed three times with PBS and were maintained in growth medium for subsequent experiments.

### 2.3. Cell Viability Assay and Flow Cytometric Analysis of Apoptosis

CCK-8 was used to measure cell viability, and HUVEC were passaged in 96-well plates. After being subjected to various treatments, the cells were incubated with 200 *μ*L of fresh culture medium containing 20 *μ*L of CCK-8 solution for 40 min at 37°C. A microplate absorbance reader (Bio-Rad) was used to measure the absorbance at a wavelength of 490 nm. Annexin V-FITC/PI double staining was used to detect cell apoptosis. HUVEC were plated in 60 mm dishes. After being exposed to different treatments, the cells were collected and washed twice with PBS and then resuspended in binding buffer at 1 × 10^6^ cells/mL. The suspension was coincubated in 5 *μ*L each of Annexin V-FITC and PI solutions for 15 min at room temperature in the dark, followed by flow cytometric analysis of apoptosis.

### 2.4. Cytoplasmic ROS and Mitochondrial ROS Detection

DCFH-DA staining was used to detect cytoplasmic ROS. DCFH-DA was diluted to a 10 *μ*mol/L final concentration with DMEM, which was applied to incubate the treated cells at 37°C for 30 min. MitoSOX Red mitochondrial superoxide indicator was used to measure mitochondrial ROS production. The indicator was diluted to a 5 *μ*mol/L final concentration with Hanks' balanced salt solution (HBSS), which was applied to incubate the treated cells at 37°C for 10 minutes. The cells were washed three times with HBSS. Fluorescent images were captured by an inverted fluorescence microscope and analyzed by Image-Pro software.

### 2.5. Mitochondrial Membrane Potential (Δ*ψ*m) Analysis

The membrane potential-specific dye JC-1 was used to detect Δ*ψ*m. After being exposed in various treatments, the cells were incubated with 0.5 *μ*mol/L JC-1 at 37°C in the dark for 15 min. After incubation, the cells were washed three times with cold PBS and imaged via florescence microscopy. At a lower Δ*ψ*m, JC-1 exists as a monomer and is visible in the green fluorescence channel. At a higher Δ*ψ*m, JC-1 accumulates as an aggregate in the mitochondrial matrix and is visible in the red channel. Therefore, a decrease in Δ*ψ*m could be detected by the transition from red fluorescence to green fluorescence.

### 2.6. Western Blotting Analysis

After various treatments, cells were collected and washed with ice-cold PBS. The cells were lysed with RIPA lysis buffer containing PMSF (1 : 100) for 30 min on ice and then centrifuged at 12000*g* for 15 min. A bicinchoninic acid (BCA) protein assay kit was used to determine the protein concentrations. Subsequently, the samples were boiled in Laemmli loading buffer. Equal 30 mg aliquots of total protein extracts were separated on an 8%, 10%, or 12% SDS-polyacrylamide gel, followed by transfer of the proteins to a polyvinylidene fluoride (PVDF) membrane. The membrane was blocked with Tris-buffered saline containing Tween 20 (TBST) supplemented with 5% nonfat dry milk for 1-2 hours at room temperature, followed by incubation with primary antibodies (1 : 1000 dilution) at 4°C for 12–24 hours. HRP-labeled secondary antibodies (1 : 5000 dilution) were applied to the membranes for an additional 2 hours at room temperature. After being washed three times with TBST, the bands were visualized using an ECL detection kit and quantified using Image Lab software.

### 2.7. Statistical Analysis

The data are presented as the means ± SD. Significant differences between treatment groups were analyzed via one-way ANOVA followed by Dunnett's test or Student's *t*-test using the Statistical Package for the Social Sciences (SPSS) 13.0 software. Statistical significance was defined as a *p*value < 0.05, <0.01, or 0.001.

## 3. Results

### 3.1. Apoptotic Effect of Hcy on HUVEC

**Figure 1:**
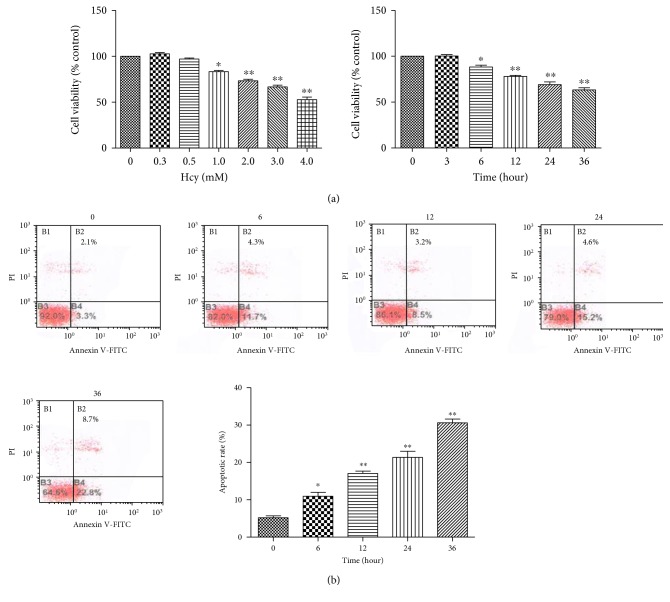
Hcy exerted an adverse effect on HUVEC viability. (a) The dose-dependent effects of Hcy treatment for 12 hours or the time-dependent effects of 2.0 mM Hcy treatment on cell viability were evaluated via CCK-8 assays (*n* = 3). (b) The apoptotic rate was assessed by Annexin V/PI double staining, and the percentage of apoptotic cells was determined based on a flow cytometric analysis. A statistical analysis of the total recorded apoptotic cells induced by Hcy at various durations was performed, and the results are shown in the bar graphs (*n* = 3). The data are presented as the means ± SD of three independent experiments. ^∗^*p* < 0.05, ^∗∗^*p* < 0.01 versus the control or 0-hour group.

The adverse effect of Hcy on HUVEC viability was analyzed by CCK-8 assays and positive staining for Annexin V-FITC/PI. HUVEC were exposed to 0, 0.3, 0.5, 1.0, 2.0, 3.0, and 4.0 mM Hcy for 12 hours. Compared with that to the 0 mM Hcy treatment, exposure to 1.0 mM Hcy for 12 hours led to a significant decrease in cell viability (Figure 1(a)). With increasing Hcy treatment concentration, cell viability decreased from 83.5% to 52.3% (Figure 1(a)). Then, we used 2.0 mM Hcy as a moderate treatment concentration. HUVEC were exposed to 2.0 mM Hcy for 0, 3, 6, 12, 24, and 36 hours. The results show that 2.0 mM Hcy led to a decrease in cell viability at 6 hours (Figure 1(a)). With prolonged exposure duration, cell viability decreased from 70.97% to 47.54% (Figure 1(a)). Similarly, total apoptotic cell percentage decreased from 81.93% to 54.55% (Figure 1(b)). These results indicated that moderate Hcy treatment decreased the viability of cardiomyocytes in a time-dependent manner.

### 3.2. Effects of Hcy on the Expression of NOX4 and the Level of Mitochondrial ROS and Cytoplasmic ROS

**Figure 2:**
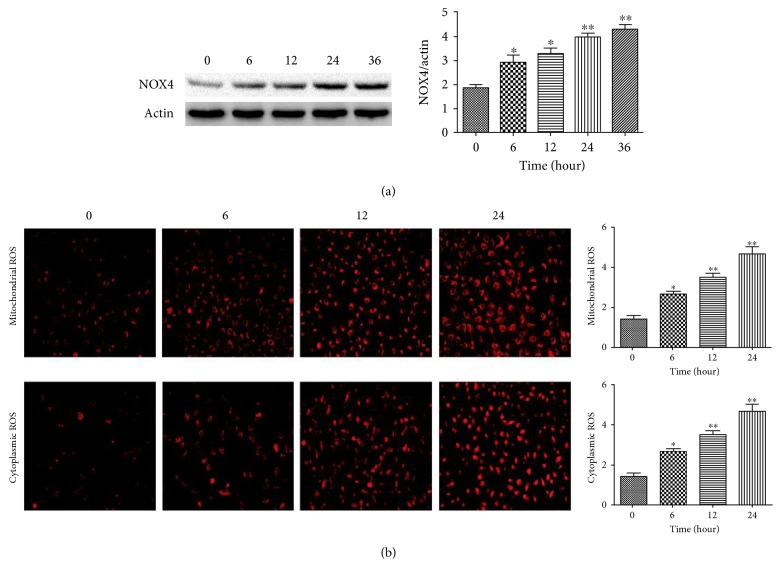
Hcy induced the expression of NOX4 and production of ROS in HUVEC in a time-dependent manner. (a) The expression levels of NOX4 were determined via Western blot analysis. *β*-actin was selected as the loading control. (b) The production of ROS in the mitochondria and cytoplasm was detected via MitoSOX Red mitochondrial superoxide indicator and DCFH-DA staining. The data are presented as the means ± SD of three independent experiments. ^∗^*p* < 0.05, ^∗∗^*p* < 0.01 versus the 0-hour group.

To explore the signaling pathways involved in Hcy-induced cellular apoptosis, HUVEC were treated with 2.0 mM Hcy for different durations, and the levels of NOX4 proteins and mitochondrial and cytoplasmic ROS were measured. The expression of NOX4 was upregulated at 6 hours after treatment, and it gradually increased with the prolonged treatment periods, peaking at 36 hours (Figure 2(a)). Similarly, Hcy treatment increased the level of mitochondrial and cytoplasmic ROS at the first 6 hours and resulted in a significantly higher level of mitochondrial and cytoplasmic ROS at 24 hours (Figure 2(b)). NOX4 is the major source of ROS in the mitochondrion, and accumulating mitochondrial ROS in turn led to mitochondrial disorder and the high level of cytoplasmic ROS.

### 3.3. Effects of Hcy on Mitochondrial Function and the Expression of p-eNOS

**Figure 3:**
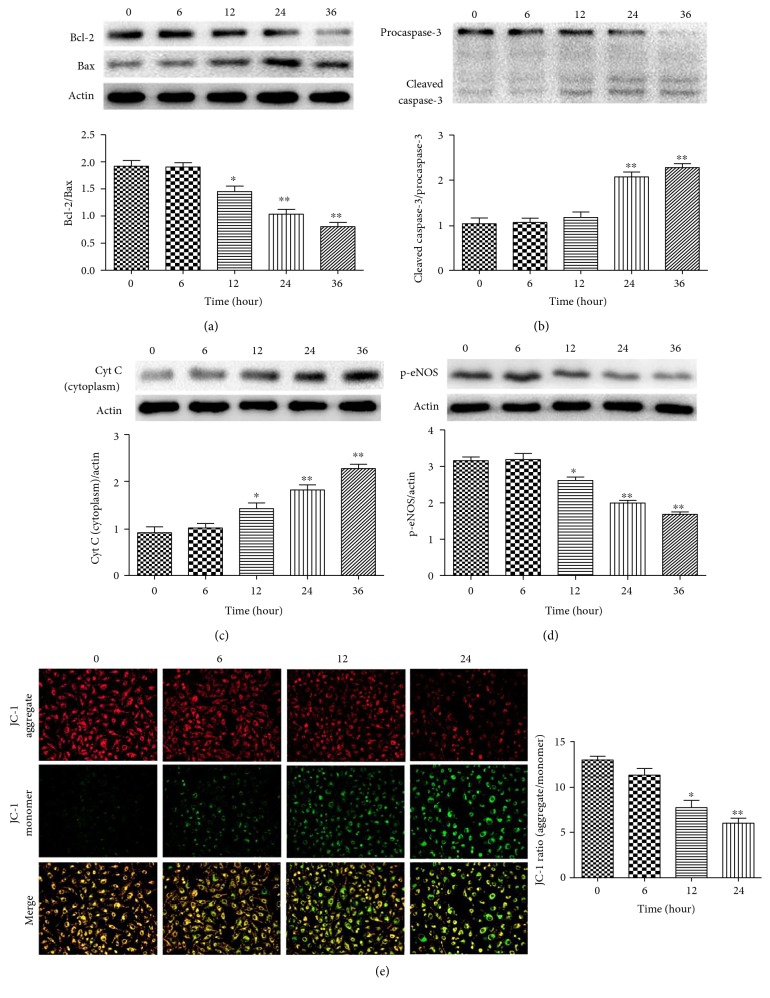
Hcy induced mitochondrial dysfunction in HUVEC in a time-dependent manner. (a, b) The time-dependent effects of Hcy on the expression of Bcl-2 and Bax were determined via Western blot analysis. *β*-actin was selected as the loading control. (b) Cleaved caspase-3 and procaspase-3 were determined via Western blot analysis. (c) The level of cytoplasmic cytochrome c was measured via Western blot analysis. *β*-actin was selected as the loading control. (d) The expression of p-eNOS was measured via Western blot analysis. *β*-actin was selected as the loading control. (e) Red indicates JC-1 aggregates, which form at a sufficient Δ*ψ*m. Green indicates JC-1 monomers, which form at a lower Δ*ψ*m. Merged images showed the colocalization of JC-1 aggregates and monomers. The data are presented as the means ± SD of three independent experiments. ^∗^*p* < 0.05, ^∗∗^*p* < 0.01 versus the control.

To gain further insight into the adverse effects of Hcy on mitochondrial function in HUVEC, we examined the mitochondrial membrane potential (Δ*ψ*m) and the expression of mitochondrial function-related proteins. After treatment with 2.0 mM Hcy, the expression of Bcl-2 was significantly decreased at 12 hours and gradually decreased with increasing treatment durations (Figure 3(a)). In contrast, the expression of Bax was markedly upregulated at 12 hours and peaked at 36 hours (Figure 3(a)). Additionally, we observed the change of mitochondrial membrane potential (Δ*ψ*m) in HUVEC exposed to Hcy. We found that the 2.0 mM Hcy treatment significantly decreased the red signal intensity while increasing the green signal intensity at 12 hours, which is indicative for the collapse of the Δ*ψ*m and the occurrence of apoptosis (Figure 3(e)). Accordingly, the level of cytoplasmic cytochrome c showed a significant increase at 12 hours (Figure 3(c)), and caspase-3 was significantly activated at 24 hours after treatment (Figure 3(b)). Furthermore, we observed a significant decrease in the level of p-eNOS at 12 hours followed by a gradual decrease until 36 hours (Figure 3(d)).

### 3.4. Effects of Hcy on ER Stress and the Activation of NF-*κ*b in HUVEC

**Figure 4:**
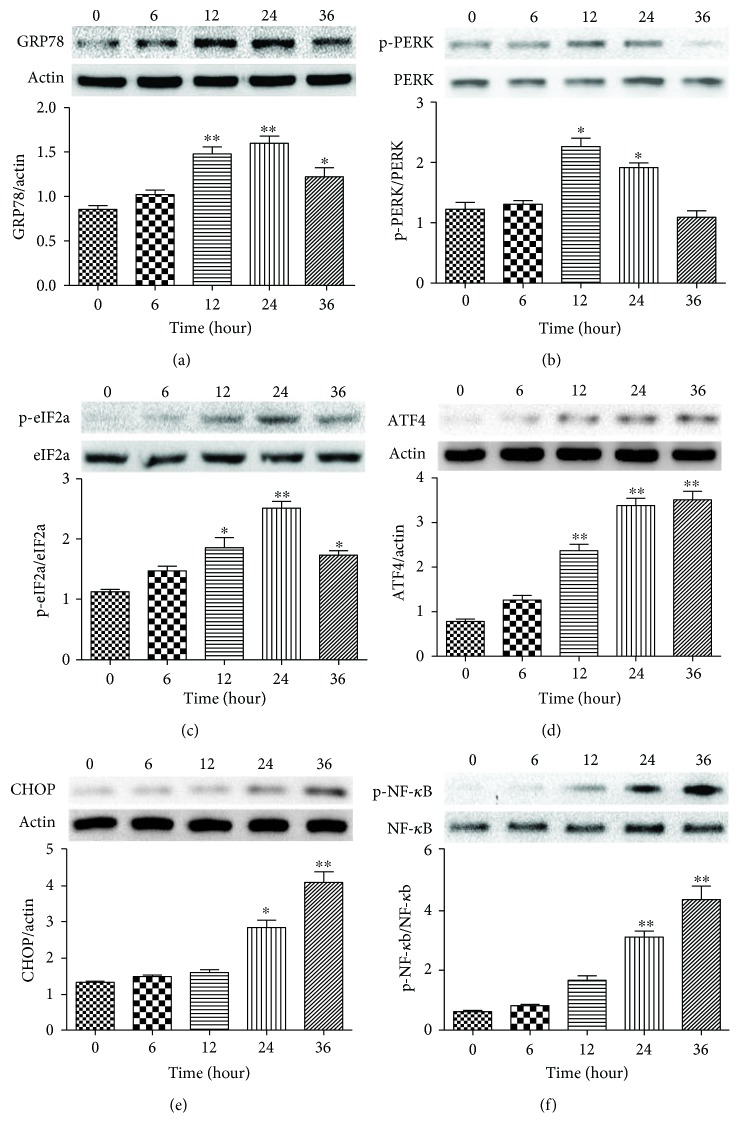
Hcy induced the expression of ER stress-related proteins in HUVEC in a time-dependent manner. (a) The expression of GRP78 was determined via Western blot analysis. *β*-actin was selected as the loading control. (b) The expression of p-PERK and PERK was determined via Western blot analysis. (c) The expression of p-eIF2a and eIF2a was determined via Western blot analysis. (d, e) The expression of ATF4 and CHOP was determined via Western blot analysis. *β*-actin was selected as the loading control. (f) The expression of p-NF-*κ*b and NF-*κ*b was determined via Western blot analysis. The data are presented as the means ± SD of three independent experiments. ^∗^*p* < 0.05, ^∗∗^*p* < 0.01 versus the 0-hour group.

Recent studies show that ER stress and subsequent molecular pathway activation are also involved in the development of HHcy-related vascular diseases. Especially, activation of the PERK signaling pathway is currently regarded as the most definitive marker of ER stress [26]. Therefore, we investigated the effects of prolonged 2.0 mM Hcy treatment on the expression of PERK signaling pathway-related proteins. 2.0 mM Hcy treatment caused a significant increase in the expression of GRP78 at 12 and 24 hours, which was then decreased at 36 hours (Figure 4(a)). PERK was markedly activated at 12 and 24 hours after treatment and then dramatically decreased at 36 hours (Figure 4(b)). The downstream protein phospho-eIF2a was increased at 12 hours, perked at 24 hours, and decreased at 36 hours (Figure 4(c)). The expression of ATF4 was markedly upregulated at 12 hours after treatment, and these elevated expression levels were sustained until 36 hours (Figure 4(d)). The expression of CHOP was significantly induced by Hcy at 24 hours and increased with the prolonged treatment duration (Figure 4(e)). Similarly, over the Hcy treatment period, the level of phospho-NF-*κ*b was slightly increased at 12 hours of Hcy treatment and significantly upregulated at 24 and 36 hours (Figure 4(f)). These observations suggest that prolonged Hcy treatment could activate the ER stress-related PERK signaling pathway.

### 3.5. Effects of NOX4 Inhibition on ER Stress and the Activation of NF-*κ*b in HUVEC

**Figure 5:**
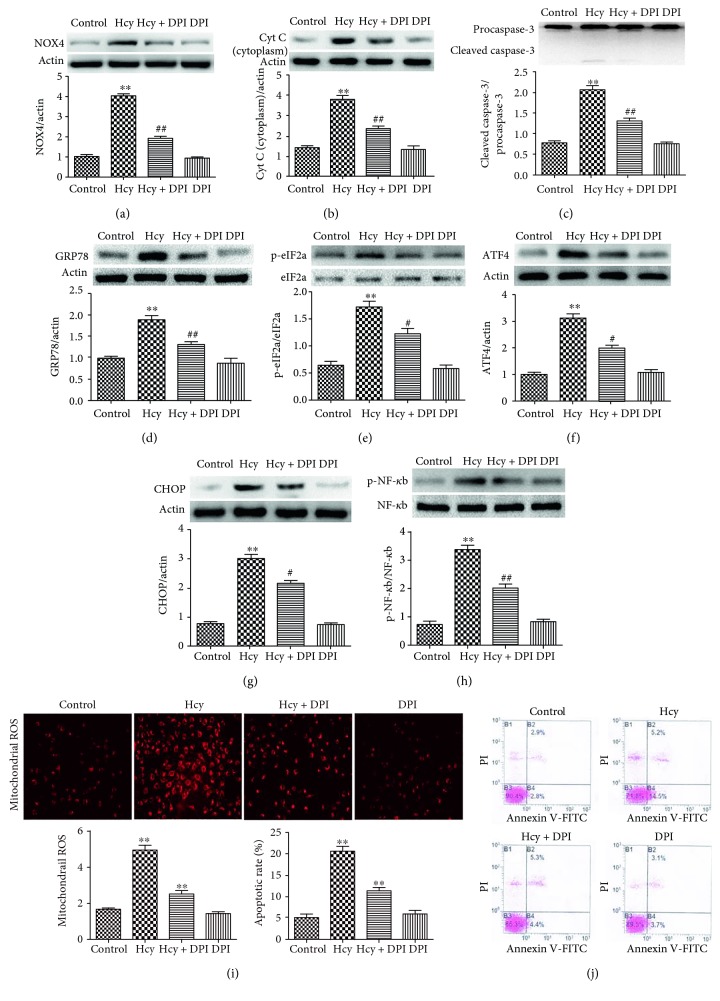
NOX4 inhibition alleviated mitochondrial dysfunction, suppressed the activation of ER stress and NF-*κ*b, and decreased apoptosis induced by Hcy in HUVEC. (a, b, c) The expression levels of NOX4, cytoplasmic cytochrome c, cleaved caspase-3, and procaspase-3 were determined via Western blot analysis. *β*-actin was selected as the loading control. (d, e, f, g) The expression of GRP78, p-eIF2a and eIF2a, ATF4, and CHOP was determined via Western blot analysis. *β*-actin was selected as the loading control. (h) The production of mitochondrial ROS was detected in different treatments groups. (j) The apoptotic rates of HUVEC exposed to different treatments were evaluated. The data are presented as the means ± SD of three independent experiments. ^∗∗^*p* < 0.01 versus the control group; ^#^*p* < 0.05, *^##^p* < 0.01 versus the Hcy group.

In this study, we examined whether the Hcy-induced mitochondrial disorder is responsible for the ER stress in HUVEC. HUVEC were pretreated with 10.0 *μ*M DPI (the NOX4 inhibitor) and were then cotreated with 2.0 mM Hcy for 24 hours. Hcy caused a significant increase in the expression of NOX4, while the high expression was inhibited by DPI pretreatment (Figure 5(a)). Therefore, we examined the mitochondrial ROS and found that the inhibition of NOX4 led to a marked decrease in mitochondrial ROS (Figure 5(i)). Furthermore, DPI pretreatment significantly decreased the level of cytoplasmic cytochrome c (Figure 5(b)), suppressed the activation of caspase-3 (Figure 5(c)), and reduced the apoptotic rate by approximately 61% (Figure 5(j)). Interestingly, DPI intervention downregulated the expression of ER stress-related proteins, which alleviated the ER stress induced by Hcy. After DPI intervention, the expression of ER stress chaperone protein GRP78 and phospho-eIF2a was marked downregulated (Figures 5(d) and 5(e)). Even the expression of ATF4 and proapoptotic CHOP proteins was obviously inhibited (Figures 5(f) and 5(g)). These observations suggest that the Hcy-induced mitochondrial disorder could modulate the activation of ER stress in HUVEC. Additionally, DPI pretreatment decreased the expression of phospho-NF-*κ*b (Figure 5(h)), suggesting that the activation of NF-*κ*b could be related to the mitochondrial disorder or ER stress.

### 3.6. The PERK Signaling Pathway Is Involved in the Activation of NF-*κ*b and Proapoptotic Effect of Hcy on HUVEC

**Figure 6:**
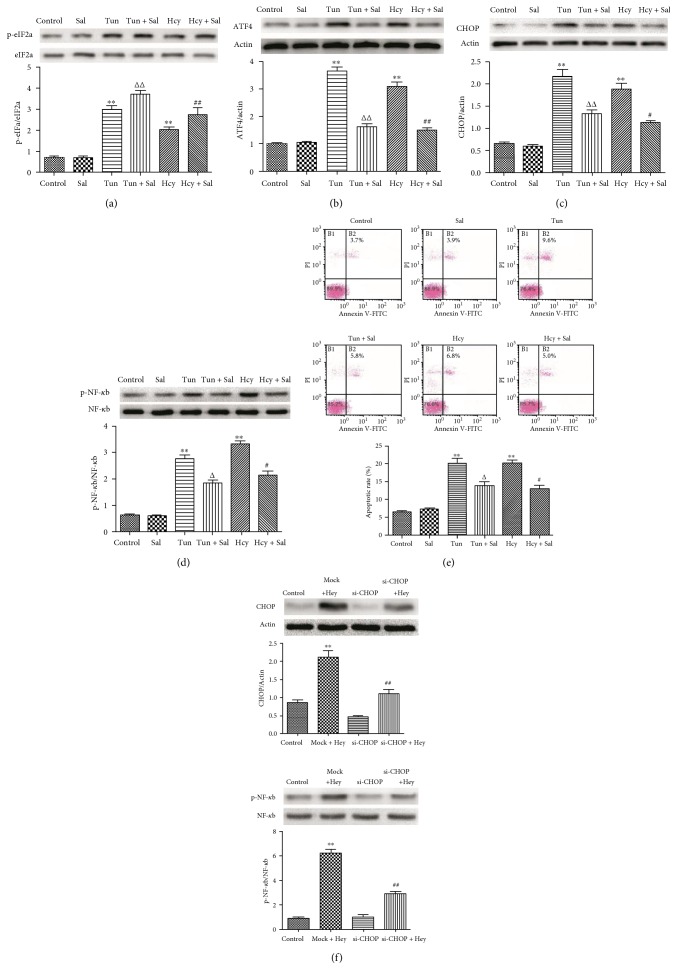
Inhibiting the PERK signaling pathway alleviated Hcy-induced apoptosis and the activation of NF-*κ*b in HUVEC. (a, b, c) The expression of p-eIF2a and eIF2a, ATF4, and CHOP was determined via Western blot analysis. *β*-actin was selected as the loading control. (d) The expression of p-NF-*κ*b and NF-*κ*b was determined via Western blot analysis. (e) The apoptotic rates of cardiomyocytes exposed to different treatments were evaluated by Annexin V/PI double staining. (f) Cells were preincubated with CHOP siRNA and then stimulated with Hcy for 24 hours. The protein levels of CHOP, p-NF-*κ*b, and NF-κb were determined via Western blot analysis. The data are presented as the means ± SD of three independent experiments. ^∗∗^*p* < 0.01 versus the control group; ^#^*p* < 0.05, *^##^p* < 0.01 versus the Hcy alone group or the mock + Hcy group; ^△^*p* < 0.05, ^△△^*p* < 0.01 versus the Tun alone group.

To investigate whether the PERK pathway is responsible for the activation of NF-*κ*b, we examined the expression of phospho-NF-*κ*b after blocking the PERK pathway using salubrinal. Salubrinal inhibits the dephosphorylation of eIF2a, maintaining its phosphorylation state [27, 28]. The high level of phospho-eIF2a can inhibit protein synthesis and enhancement of the protein folding capacity of the ER, which blocks the expression of proapoptotic protein CHOP. Therefore, salubrinal specifically blocks the PERK signaling pathway. Furthermore, tunicamycin, the specific stimulator of ER stress, as the positive control, was used to induce ER stress in HUVEC. The results showed that the dephosphorylation of eIF2a induced by Hcy and tunicamycin obviously decreased following the intervention of salubrinal (Figure 6(a)). The expression of ATF4 and CHOP, as well as phospho-NF-*κ*b, can be induced by 2.0 mM Hcy or 4.0 *μ*M tunicamycin treatments, while the expression of these proteins can be markedly downregulated by salubrinal intervention (Figures 6(b), 6(c), and 6(d)). Annexin V/PI double staining indicated that thapsigargin or Hcy induced apoptosis in 18.3% or 17.6% of all cells but that salubrinal intervention decreased the apoptotic rate to 12.7% or 11.2% (Figure 6(e)). It is suggested that the PERK signaling pathway could be involved in modulating the activation of NF-*κ*b. Therefore, to further explore the role of the PERK signaling pathway in the activation of NF-*κ*b, transfection of HUVEC with a specific siRNA against the CHOP gene was performed. After preincubation with CHOP siRNA and then stimulation with 2.0 mM Hcy for 24 hours, the protein expression levels of CHOP were significantly downregulated compared with those of the mock + Hcy group (Figure 6(f)). The expression of phospho-NF-*κ*b was significantly decreased following the downregulation of CHOP protein (Figure 6(f)). These observations suggested that the PERK signaling pathway might be one of the important pathways modulating the activation of NF-*κ*b.

## 4. Discussion

**Figure 7:**
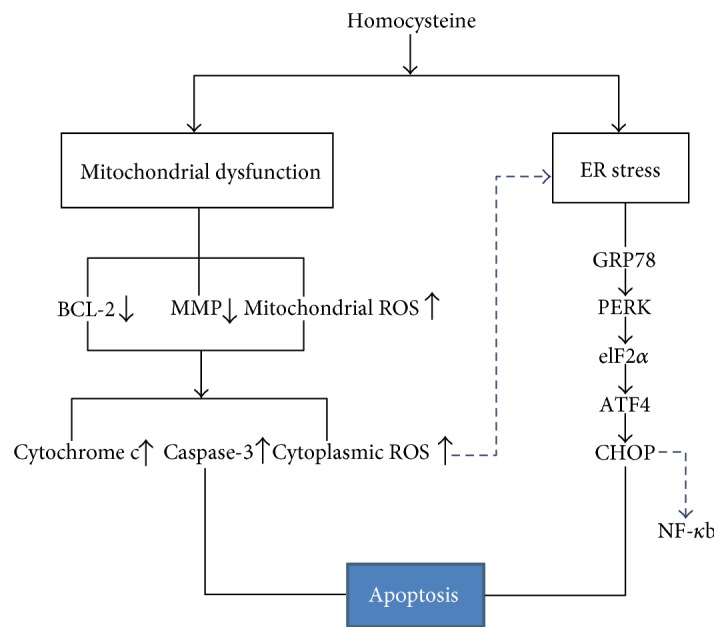
The diagram of the proposed pathway where Hcy exerts its adverse effect on mitochondrial function and ER stress of the HUVEC. The diagram shows signal transduction events associated with Hcy-induced mitochondrial dysfunction and ER stress. Hcy induces mitochondrial dysfunction including decreased expression of Bcl-2 and MMP and increased mitochondrial ROS causing an increase in the levels of cytoplasmic cytochrome c, cytoplasmic ROS, and caspase-3, which crucially results in HUVEC apoptosis. Simultaneously, Hcy increases expression of GRP78, activates PERK, causes phosphorylation of eIF2*α*, and finally induces expression of ATF4 and CHOP, thereby partly contributing to HUVEC apoptosis and modulating the activation of NF-*κ*b. In addition, the accumulated cytoplasmic ROS may cause the production of misfolded proteins, thereby triggering the ER stress of HUVEC.

Hcy-induced endothelial cell apoptosis is known to contribute to the development of vascular disease [29, 30]. The proposed molecular and cellular mechanisms underlying the pathological process are not well understood. In this study, to clarify the precise and detailed mechanisms underlying the opposing effects of Hcy on HUVEC, in vitro experiments were performed. We demonstrated that moderate Hcy concentration treatment can induce apoptosis of HUVEC in a time-dependent manner and that this noxious effect of Hcy was related to mitochondrial dysfunction and ER stress. The signal transduction events associated with Hcy-induced mitochondrial dysfunction and ER stress are described in Figure 7. Hcy-induced mitochondrial dysfunction mainly modulated apoptosis of HUVEC and might be responsible for the initiation of ER stress. Prolonged ER stress resulted in the excessive activation of the PERK pathway, which could be involved in moderating the apoptosis of HUVEC and the activation of NF-*κ*b.

Atherosclerosis is a complex, chronic process that is initiated at sites of endothelial cell injury. Injured endothelial cells can cause impairment of endothelium-dependent relaxation of blood vessels, thereby resulting in vascular dysfunction. Both preclinical and clinical studies have shown that Hcy is a powerful independent stimulus that promotes the development of cardiovascular diseases including atherosclerosis and hypertension [31–33]. In severe HHcy, circulating endothelial cells have been detected, indicative of endothelial cell death. However, at present, we have limited knowledge of the molecular and cellular mechanisms responsible for Hcy-induced endothelial cell apoptosis. In our study, the results show that the moderate Hcy concentration induced a significant increase in apoptosis and a marked decrease in the expression in p-eNOS in HUVEC, provided that prolonging the treatment duration was performed. Our findings further validated the noxious effects of Hcy on endothelial cells, which could be related to the stimulating duration. Therefore, moderately elevated plasma Hcy levels also deserve our attention because this condition is highly prevalent in the general population and is potentially an important risk factor for the exacerbation of vascular injury. However, this condition is often ignored.

In previous studies, Hcy has been established to induce higher levels of intracellular ROS, which contributed to cellular injury and vascular dysfunction [34–36]. Nevertheless, these studies seldom investigated the source of intracellular ROS and the relationship with HUVEC death. In mammals, NOX families are important sources of ROS. As one of the members of NOX families, NOX4 is unique as it is predominantly localized in the mitochondria, has constitutive activity, and results in the production and accumulation of mitochondrial ROS [37, 38]. The present study showed that Hcy could upregulate the expression of NOX4 in a time-dependent manner, which led to the production of mitochondrial ROS, suggesting that the Hcy-induced upregulation of NOX4 could be the important sources of intracellular ROS in HUVEC. Additionally, in our study, with the accumulating mitochondrial ROS, the ratio of Bcl-2/Bax and MMP was decreased and the leakage of cytochrome c and HUVEC apoptosis was increased, whereas these conditions were significantly improved following the decreased production of mitochondrial ROS. On the basis of these observations, we suggested that excessive mitochondrial ROS production could decrease the expression of Bcl-2, which caused the change of mitochondrial membrane permeabilization, thereby resulting in HUVEC apoptosis. At present, there is a causal relationship between HHcy, endothelial dysfunction, and accelerated atherosclerosis in animal models of genetic- and diet-induced HHcy [39, 40]. However, the major proposed molecular and cellular mechanisms underlying the atherogenic effects of HHcy are incompletely understood. Our findings provide direct evidence that mitochondrial dysfunction induced by prolonged Hcy treatment crucially contributed to apoptotic endothelial cell death, which might be the important mechanism by which Hcy induces atherosclerosis. Furthermore, Xu et al. have reported that Hcy induces apoptosis in HUVEC which is accompanied by an increased level of caspase-3 expression and activation [41]. Under different physiological conditions, caspase-3 is usually considered an exe-apoptotic protein, whereas the proposed molecular signaling mechanisms modulating caspase-3 are various. In our study, prolonged Hcy treatment obviously increased the expression of cleaved caspase-3 in HUVEC, which was in accordance with the previous study. Our study further showed that the activation of caspase-3 was obviously suppressed following the improved mitochondrial function, suggesting that the activation of caspase-3 could result from the mitochondrial disorder in Hcy-induced HUVEC.

Prolonged or severe ER stress can result in apoptotic cell death and contribute to the pathogenesis of a number of human diseases, including atherosclerosis, hypertension, and left ventricular hypertrophy [42]. Previous studies demonstrated that Hcy is one of the ER stress inducers on endothelial cells based on observations that it could cause the induction of ER chaperones [43]. However, the molecular and cellular mechanisms explaining how Hcy induces ER stress and the direct effect of ER stress on HUVEC apoptosis have not been defined yet. Indeed, we found that Hcy upregulated the expression of GRP78 and activated the PERK, suggesting that Hcy initiates ER stress in HUVEC. Nevertheless, more importantly, great attention should be paid to the complete signaling pathway of ER stress if we want to better understand the relationship between ER stress and Hcy-induced apoptosis. Furthermore, the time course of the onset of ER stress should be consistent with the process of cell apoptosis if apoptosis results from ER stress. Therefore, we studied the functional effect of the PERK-eIF2*α*-ATF4-CHOP signaling pathway on HUVEC apoptosis in a time-dependent manner. Activation of the PERK pathway is currently regarded as the most definitive marker of ER stress. As one of the ER resident chaperones, PERK can be activated by the accumulation of misfolded proteins, resulting in the phosphorylation of eIF2*α*. The phosphorylation of eIF2*α* blocks its ability to function as an initiator of translation leading to a halt in protein synthesis. The findings presented in this study indicated that Hcy activated the PERK and induced the phosphorylation of eIF2*a* at the early periods, suggesting that excessively misfolded protein accumulation could be the reason explaining the activation of the ER stress in HUVEC exposed to Hcy. The results also showed that the prolonged Hcy treatment significantly upregulated the expression of ATF4 and subsequently induced the expression of proapoptotic protein CHOP, suggesting that prolonged Hcy treatment could excessively activate the PERK signaling pathway. To investigate the functional role of the PERK signaling pathway in Hcy-induced HUVEC apoptosis, salubrinal was specially used to inhibit the dephosphorylation of eIF2a, thereby blocking the PERK signaling pathway. The results indicated that the inhibition of the PERK pathway obviously alleviated HUVEC apoptosis, suggesting that the PERK signaling pathway could be involved in modulating HUVEC apoptosis. Thus, in addition to causing endothelial injury directly by mitochondrial dysfunction, Hcy also induces the excessive activation of ER stress and thereby potentiates endothelial cell death; this may be helpful for explaining why Hcy contributes to atherosclerosis and essential hypertension. Furthermore, we investigated how Hcy treatment initiated ER stress in HUVEC. Increasing evidence has demonstrated that there are various factors contributing to the production of misfolded proteins, while higher intracellular ROS levels could be one of the most direct triggers [44, 45]. Our results indicated that inhibiting the production of intracellular ROS could ameliorate Hcy-induced ER stress in HUVEC, thereby suggesting that the increasing intracellular ROS might be responsible for the accumulated misfolded proteins and activation of ER stress in Hcy-induced HUVEC. Taken together, our findings provide direct evidence that the excessive activation of the PERK signaling pathway could modulate Hcy-induced HUVEC apoptosis. Under pathological conditions of HHcy, the production of intracellular ROS could contribute to the accumulated misfolded proteins in ER, thereby initiating the ER stress of HUVEC. That might be the bridge linking mitochondrial dysfunction and the activation of ER stress.

Local inflammatory response is recognized as an important mechanism leading to vascular dysfunction and subsequent injury [46, 47]. Many cellular signaling transduction pathways and cytokines have been demonstrated to be related to the process of the inflammatory response, but the exact mechanism is still not comprehended in Hcy-induced HUVEC. In this context, it is interesting to note that prolonged Hcy treatment induced the activation of NF-*κ*b-mediated inflammation. This evidence motivated us to explore the molecular mechanisms modulating the activation of NF-*κ*b. We observed that blocking the PERK signaling pathway and si-CHOP effectively suppressed the activation of NF-*κ*b, indicating that the excessive activation of the PERK pathway could be one of the cellular signaling transduction pathways modulating the activation of NF-*κ*b in Hcy-induced HUVEC. Nevertheless, we cannot exclude the involvement of other molecular mechanisms in the activation of NF-*κ*b.

In summary, as shown in Figure 7, the present results suggest that Hcy-induced mitochondrial dysfunction is the crucial molecular mechanism mediating HUVEC apoptosis. Additionally, Hcy-induced mitochondrial dysfunction could be responsible for the activation of the PERK signaling pathway, which contributed to the occurrences of apoptosis and inflammation. Our findings may be beneficial for understanding the complex molecular mechanisms underlying Hcy-related atherosclerosis and hypertension.

## References

[1] Malinow M. R., Bostom A. G., Krauss R. M. (1999). Homocyst(e)ine, diet, and cardiovascular diseases: a statement for healthcare professionals from the Nutrition Committee, American Heart Association. *Circulation*.

[2] Marasini B., Massarotti M., Biondi M. L. (2002). Homocysteine and vascular diseases. *Circulation*.

[3] Thambyrajah J., Townend J. N. (2000). Homocysteine and atherothrombosis - mechanisms for injury. *European Heart Journal*.

[4] Kim Y. W., West X. Z., Byzova T. V. (2013). Inflammation and oxidative stress in angiogenesis and vascular disease. *Journal of Molecular Medicine*.

[5] Kim Y. W., Byzova T. V. (2014). Oxidative stress in angiogenesis and vascular disease. *Blood*.

[6] Xie Y. W., Wolin M. S. (1996). Role of nitric oxide and its interaction with superoxide in the suppression of cardiac muscle mitochondrial respiration. *Involvement in Response to Hypoxia/Reoxygenation. Circulation*.

[7] Handy D. E., Zhang Y., Loscalzo J. (2005). Homocysteine downregulates cellular glutathione peroxidase (GPx1) by decreasing translation. *The Journal of Biological Chemistry*.

[8] Wang X., Cui L., Joseph J. (2011). Homocysteine induces cardiomyocyte dysfunction and apoptosis through p38 MAPK-mediated increase in oxidant stress. *Journal of Molecular and Cellular Cardiology*.

[9] Okada K., Minamino T., Tsukamoto Y. (2004). Prolonged endoplasmic reticulum stress in hypertrophic and failing heart after aortic constriction: possible contribution of endoplasmic reticulum stress to cardiac myocyte apoptosis. *Circulation*.

[10] Ivanova E. A., Orekhov A. N. (2016). The role of endoplasmic reticulum stress and unfolded protein response in atherosclerosis. *International Journal of Molecular Sciences*.

[11] Wei H., Zhang R., Jin H., Liu D., Tang X., Tang C. (2010). Hydrogen sulfide attenuates hyperhomocysteinemia-induced cardiomyocytic endoplasmic reticulum stress in rats. *Antioxidants & Redox Signaling*.

[12] Lentz S. R., Sadler J. E. (1991). Inhibition of thrombomodulin surface expression and protein C activation by the thrombogenic agent homocysteine. *The Journal of Clinical Investigation*.

[13] Wang X. C., Sun W. T., Yu C. M. (2015). ER stress mediates homocysteine-induced endothelial dysfunction: modulation of IKCa and SKCa channels. *Atherosclerosis*.

[14] Dionisio N., Jardín I., Salido G. M., Rosado J. A. (2015). Homocysteine, intracellular signaling and thrombotic disorders. *Current Medicinal Chemistry*.

[15] Fu H. Y., Okada K., Liao Y. (2010). Ablation of C/EBP homologous protein attenuates endoplasmic reticulum-mediated apoptosis and cardiac dysfunction induced by pressure overload. *Circulation*.

[16] Wang M., Kaufman R. J. (2016). Protein misfolding in the endoplasmic reticulum as a conduit to human disease. *Nature*.

[17] Quick Q. A., Faison M. O. (2012). CHOP and caspase 3 induction underlie glioblastoma cell death in response to endoplasmic reticulum stress. *Experimental and Therapeutic Medicine*.

[18] Oyadomari S., Yun C., Fisher E. A. (2006). Cotranslocational degradation protects the stressed endoplasmic reticulum from protein overload. *Cell*.

[19] Liu M. Q., Chen Z., Chen L. X. (2016). Endoplasmic reticulum stress: a novel mechanism and therapeutic target for cardiovascular diseases. *Acta Pharmacologica Sinica*.

[20] Luoma P. V. (2013). Elimination of endoplasmic reticulum stress and cardiovascular, type 2 diabetic, and other metabolic diseases. *Annals of Medicine*.

[21] Han S., Wu H., Li W., Gao P. (2015). Protective effects of genistein in homocysteine-induced endothelial cell inflammatory injury. *Molecular and Cellular Biochemistry*.

[22] Wang D., Wang H., Luo P. (2012). Effects of ghrelin on homocysteine-induced dysfunction and inflammatory response in rat cardiac microvascular endothelial cells. *Cell Biology International*.

[23] Mangge H., Becker K., Fuchs D., Gostner J. M. (2014). Antioxidants, inflammation and cardiovascular disease. *World Journal of Cardiology*.

[24] Dionisio N., Jardín I., Salido G. M., Rosado J. A. (2010). Homocysteine, intracellular signaling and thrombotic disorders. *Current Medicinal Chemistry*.

[25] Carluccio M. A., Ancora M. A., Massaro M. (2012). Homocysteine induces VCAM-1 gene expression through NF-kappaB and NAD(P)H oxidase activation: protective role of Mediterranean diet polyphenolic antioxidants. *American Journal of Physiology. Heart and Circulatory Physiology*.

[26] Shi K., Wang D., Cao X., Ge Y. (2013). Endoplasmic reticulum stress signaling is involved in mitomycin C (MMC)-induced apoptosis in human fibroblasts via PERK pathway. *PloS One*.

[27] He L., Lee J., Jang J. H. (2013). Osteoporosis regulation by salubrinal through eIF2alpha mediated differentiation of osteoclast and osteoblast. *Cellular Signalling*.

[28] Matsuoka M., Komoike Y. (2015). Experimental evidence shows salubrinal, an eIF2alpha dephosphorylation inhibitor, reduces xenotoxicant-induced cellular damage. *International Journal of Molecular Sciences*.

[29] Bertoia M. L., Pai J. K., Cooke J. P. (2015). Plasma homocysteine, dietary B vitamins, betaine, and choline and risk of peripheral artery disease. *Atherosclerosis*.

[30] Kwon H. M., Lee Y. S., Bae H. J., Kang D. W. (2014). Homocysteine as a predictor of early neurological deterioration in acute ischemic stroke. *Stroke*.

[31] Naghshtabrizi B., Shakerian F., Hajilooi M., Emami F. (2012). Plasma homocysteine level and its genotypes as a risk factor for coronary artery disease in patients undergoing coronary angiography. *J Cardiovasc Dis Res*.

[32] Ji Y., Tan S., Xu Y. (2013). Vitamin B supplementation, homocysteine levels, and the risk of cerebrovascular disease: a meta-analysis. *Neurology*.

[33] Mehlig K., Leander K., de Faire U. (2013). The association between plasma homocysteine and coronary heart disease is modified by the MTHFR 677C>T polymorphism. *Heart*.

[34] Papatheodorou L., Weiss N. (2007). Vascular oxidant stress and inflammation in hyperhomocysteinemia. *Antioxidants & Redox Signaling*.

[35] Dong F., Zhang X., Li S. Y., Zhang Z., Ren Q., Culver B. (2005). Possible involvement of NADPH oxidase and JNK in homocysteine-induced oxidative stress and apoptosis in human umbilical vein endothelial cells. *Cardiovascular Toxicology*.

[36] Topal G., Brunet A., Millanvoye E. (2004). Homocysteine induces oxidative stress by uncoupling of NO synthase activity through reduction of tetrahydrobiopterin. *Free Radical Biology & Medicine*.

[37] Kim S. H., Kim K. Y., Yu S. N. (2016). Silibinin induces mitochondrial NOX4-mediated endoplasmic reticulum stress response and its subsequent apoptosis. *BMC Cancer*.

[38] Piccoli C., Ria R., Scrima R. (2005). Characterization of mitochondrial and extra-mitochondrial oxygen consuming reactions in human hematopoietic stem cells. Novel evidence of the occurrence of NAD(P)H oxidase activity. *The Journal of Biological Chemistry*.

[39] Wang H., Jiang X., Yang F. (2003). Hyperhomocysteinemia accelerates atherosclerosis in cystathionine betasynthase and apolipoprotein E double knock-out mice with and without dietary perturbation. *Blood*.

[40] Hofmann M. A., Lalla E., Lu Y. (2001). Hyperhomocysteinemia enhances vascular inflammation and accelerates atherosclerosis in a murine model. *The Journal of Clinical Investigation*.

[41] Xu Z., Lu G., Wu F. (2009). Simvastatin suppresses homocysteine-induced apoptosis in endothelial cells: roles of caspase-3, cIAP-1 and cIAP-2. *Hypertension Research*.

[42] Ni M., Lee A. S. (2007). ER chaperones in mammalian development and human diseases. *FEBS Letters*.

[43] Andrew Outinen P., Sood S. K., Pfeifer S. I. (1999). Homocysteine-induced endoplasmic reticulum stress and growth arrest leads to specific changes in gene expression in human vascular endothelial cells. *Blood*.

[44] Yin L., Dai Y., Cui Z., Jiang X., Liu W., Han F. (2016). The regulation of cellular apoptosis by the ROS-triggered PERK/EIF2α/chop pathway plays a vital role in bisphenol A-induced male reproductive toxicity. *Toxicology and Applied Pharmacology*.

[45] Li L., Zhang Q. G., Lai L. Y. (2016). Neuroprotective effect of ginkgolide B on bupivacaine-induced apoptosis in SH-SY5Y cells. *Oxidative Medicine and Cellular Longevity*.

[46] Potente M., Gerhardt H., Carmeliet P. (2011). Basic and therapeutic aspects of angiogenesis. *Cell*.

[47] Sibley C. T., Estwick T., Zavodni A. (2015). Assessment of atherosclerosis in chronic granulomatous disease. *Circulation*.

